# Effects of Phenolic-Rich Extracts from *Castanea sativa* Mill. Wood Processing Byproducts on the Development of Compostable Polylactic Acid-Based Materials with Antioxidant and Antibacterial Properties

**DOI:** 10.1021/acsomega.5c09394

**Published:** 2026-01-15

**Authors:** Andrea Lombardi, Franco Dominici, Margherita Campo, Pamela Vignolini, Andrea Fochetti, Mirco Pizzetti, Marco Papalini, Francesca Luzi, Roberta Bernini, Debora Puglia

**Affiliations:** a Department of Agriculture and Forest Sciences (DAFNE), 19054 University of Tuscia, 01100 Viterbo, Italy; b Bioricerche S.r.l., Loc. Ferro di Cavallo, 58034 Castell’Azzara, Italy; c Civil and Environmental Engineering Department, 9309 University of Perugia, Strada di Pentima 4, 05100 Terni, Italy; d Phytolab, Department of Statistics, Informatics, Applications “G. Parenti”, DiSIA, University of Florence, Via Ugo Schiff 6, 50019 Sesto Fiorentino, Italy; e Department of Science and Engineering of Matter, Environment and Urban Planning (SIMAU), 9294 Polytechnic University of Marche, Via Brecce Bianche 12, 60131 Ancona, Italy

## Abstract

In a circular economy framework, valorizing agro-industrial waste into high-performance active materials is crucial for sustainable packaging and biomedical innovative applications. This study reported the design, development, and characterization of multifunctional polylactic acid (PLA)-derived films obtained by extrusion, incorporating PLA with an extract from *Castanea sativa* Mill. wood (CSW) by-products. The phenolic profile of the extract was identified through HPLC-DAD-MS analysis, highlighting the presence of gallic acid, ellagic acid, and hydrolyzable tannins as vescalagin and castalagin. TGA analysis evidenced thermal stability till 250 °C according to the extrusion process. Interestingly, CSW extract exhibited *in vitro* antimicrobial properties against Gram-positive relevant bacterial strains (MBC from 0.5% to 1.0% w/v). Finally, active films (PLA_1CSW, PLA_3CSW, and PLA_5CSW) were prepared by extruding PLA with CSW extract at 1, 3, and 5%, w/w, respectively. Mechanical characterization of these films showed that tensile strength and modulus remained comparable to neat PLA, while elongation at break decreased with increasing CSW content. Antioxidant assays showed dose-dependent DPPH radical scavenging activity, reaching a value of 96% for PLA_3CSW. PLA_5CSW revealed strong antimicrobial performance against *Staphylococcus aureus* and *Bacillus cereus*. For all formulations, overall migration into food simulant remained below EU limits (<10 mg/dm^2^) and, according to ISO 20200:2016, disintegration under controlled composting conditions reached >90% within 14 days. These findings revealed the potential of CSW extract in the production of sustainable, compostable, and bioactive PLA-based films, according to a sustainable transition following circular economy principles in the food and biomedical sectors.

## Graphical abstract

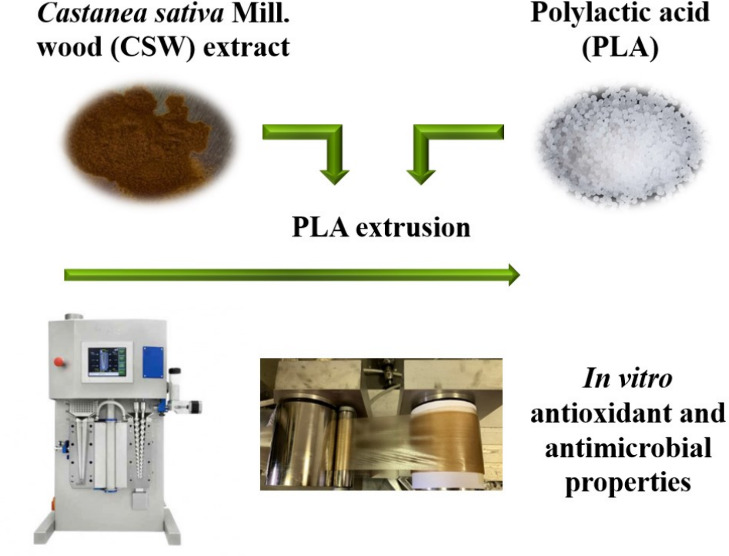

## 1 Introduction

In recent years, the scientific and industrial communities have been exploring sustainable solutions to reduce dependence on plastic and fossil-based materials. To date, the plastic industry is still dominated by high-energy and polluting processes, with above 90% of production derived from fossil fuels.^1^ The inefficiency of reusing and recycling systems provides this economic sector with one of the main contributors to the triple planetary crisis. At the same time, in addition to being large users of plastic packaging, the agro-food chain generates significant amounts of waste biomass from the early stages of industrial production.^2^ These challenges, from the accumulation of nondegradable plastics to the depletion of fossil resources and the related emissions and climate intakes, highlight the urgency of transition toward production models compatible with the principle of the circular economy.^3^

A promising response is offered by biopolymers, which are able to counteract the use of plastics and minimize the associated environmental impact. Recent advances in biomass fermentation and synthetic technologies fostered the production of biobased bioplastics, increasing the potential of these materials to achieve a transition to circular economic models.^4^ In this scenario, polylactic acid (PLA), an aliphatic polyester obtained from the polymerization of lactic acid, stands out for its compatibility with traditional processing technologies, good thermoplastic properties, and compostability under industrial conditions.^5^ To date, PLA-based bioplastics are diffused in a wide range of industrial sectors, from food packaging to single-use devices and textiles, cosmetics, and automotive.^6^ However, large-scale adoption of biopolymers is slowed down by economic, technological, and infrastructural barriers. In addition, the shortage of dedicated raw materials, the lack of incentives for enterprises, and the high degree of specialization involved in the production process for conventional plastics represent further obstacles to the transition.^7^

In this context, a noteworthy research line involves the design and development of innovative active materials able to combine biodegradability, renewable raw materials, and advanced properties, such as antioxidant and/or antimicrobial activity, enhancing the added value of bioplastics, especially in high-intensity industrial sectors.^8^ To increase the sustainability of active materials, both natural organic products and extracts have been investigated as potential ingredients for the development of innovative materials.^9–11^ Among them, phenolic compounds represent key structures due to their antioxidant, antimicrobial, and chelating properties.^12^ Widespread in plants, they can also be found and recovered in exploitable concentrations from agro-industrial wastes and byproducts.^13,14^

Tannins are natural phenolic macromolecules employed in several industrial processes and, due to their ability to interact with other molecules, are suitable compounds for materials development.^15^ Sweet chestnut (*Castanea sativa* Mill.) is the most common chestnut species in Europe, mainly cultivated in the southern region for fruit and wood production.^16^ Historically known for its high nutritional properties, in the last decades, it has attracted the attention of academics and industrial stakeholders for its considerable amount of tannins.^17^ In detail, this species is particularly rich in gallic acid and hydrolyzable tannins as castalagin and vescalagin, responsible for a wide range of *in vitro* and *in vivo* biological activities.^18–20^ Furthermore, waste and byproducts from cultivation and processing represent abundant, underutilized, and low-cost resources whose valorization through green extraction technologies is aligned with circular economy principles.^21,22^ Some studies started to explore the integration of chestnut extracts into polymeric and biopolymeric matrices, revealing promising structural and functional properties.^23^ However, systematic evidence is still limited.^11^ Moreover, the lack of data concerning the compostability of active materials with natural extracts acts as a critical issue to assess the compliance with biological disposal systems and prevent technical “greenwashing” phenomena.

In this study, a phenolic-rich extract was obtained from *Castanea sativa* Mill. wood (CSW), obtained through a sustainable industrial process and characterized by HPLC-DAD-MS analysis for phenolic content, was utilized as an active ingredient in developing a compostable PLA-based active film through extrusion. The objective was to assess the compatibility of this extract with PLA film processing techniques, evaluate the impact of different extract loading concentrations on structural, mechanical, thermal, antioxidant, antimicrobial properties, and disintegration under industrial composting conditions.

## 2 Materials and Methods

### 2.1 Samples and Reagents

All solvents for HPLC-DAD-MS analyses (LC-MS grade) and formic acid (analytical grade) were purchased from Sigma Aldrich Chemical Co., Inc. (Milwaukee, WI, USA). Gallic acid and ellagic acid were supplied by Extrasynthèse S.A. (Lyon, Nord-Genay, France). *Castanea sativa* Mill. Wood (CSW) extract was commercialized by Gruppo Mauro Saviola Srl (Viadana, Italy). PLA 4032D grade (average molecular weight of 210 kg/mol and a melt flow index of 7 g/10 min (210 °C, 2.16 kg)) was purchased from NatureWorks.

### 2.2 HPLC-DAD-MS Analysis of CSW Extract

HPLC-DAD-MS analysis was performed by using an HP-1260 liquid chromatograph equipped with a DAD detector and an MSD API-electrospray (Agilent Technologies, Santa Clara, CA, USA) operating in negative ionization mode. Mass spectrometer operating conditions were the following: gas temperature of 350 °C, flow rate of 10.0 L/min, nebulizer pressure of 30 psi, quadrupole temperature of 30 °C, and capillary voltage of 3500 V. The fragmentor was set at 120 eV. A Luna column, C18 250 × 4.60 mm, 5 μm column (Phenomenex, Torrance, CA, USA), operating at 25 °C, was used. The eluents were H_2_O (adjusted to pH 3.2 with HCOOH) and CH_3_CN. A four-step linear solvent gradient starting from 100% H_2_O up to 100% CH_3_CN was performed with a flow rate of 0.8 mL/min over a 55 min period, as previously described.^21^ The polyphenolic compounds were identified according to their chromatographic, spectrophotometric, and spectrometric data by comparing their retention times and HPLC-DAD and HPLC-MS data with those of the available specific commercial standards, also considering previous results and literature data. Quantitative analysis was performed by HPLC-DAD using a five-point regression curve. Determinations of the polyphenol content were carried out in triplicate; the results are given as means with respective standard deviations.

### 2.3 Determination of the Minimal Bactericidal Concentration (MBC) of CSW Extract against Different Bacterial Strains

The MBC of the CSW extract was evaluated against both Gram–negative and Gram-positive bacterial strains using a dilution method. A microbial suspension was obtained from the revitalization of the cryovials. Once optimal growth was achieved, an aliquot of the suspension was collected and standardized to 0.5 McFarland density (approximately 10^8^ colony-forming units/mL or CFU mL^–1^) to produce the inoculum solution. Subsequently, 1 mL of this inoculum was added to a sterile test tube with 9 mL of a CSW solution at different concentrations. Test tubes were incubated at the specific conditions required for each target organism for 24 h. Following incubation, the tube content was cultured on a microorganism-specific solid agar. Antimicrobial activity was assessed based on the absence of growth and correlated with MBC. All of the determinations were performed in triplicate.

### 2.4 Production of PLA-based films (PLA_1CSW, PLA_3CSW, and PLA_5CSW)

Before processing, PLA granules were dried at 80 °C and the extract powder at 40 °C for 4 h to reduce the moisture content and avoid hydrolysis phenomena during extrusion. Biocomposite films based on PLA added with CSW extract, and the reference matrix of PLA were processed by a corotating twin screw microextruder (Xplore 5 & 15 Micro Compounder, Sittard, The Netherlands). Films with a width of approximately 60 mm and nominal thickness ranging from 30 to 60 μm were obtained by coupling the extruder with a 65 mm Xplore Film Device and using a temperature-controlled cast film die. The temperature profile was set at 170 – 175 – 180 °C in the three heating zones of the extruder, mixing for 3 min at a screw speed of 90 rpm. Die temperature was set at 185 °C, the film line at a drawing speed of 600 mm·min^–1^ of the draw-off roller and a winding torque of 30 N·mm for the take-up roller. Samples of neat PLA and biocomposite films with 1 wt % (PLA_1CSW), 3 wt % (PLA_3CSW), and 5 wt % (PLA_5CSW) of CSW extract were obtained.

### 2.5 Thermal Characterization

Thermogravimetric analysis (TGA, Seiko Exstar 6300) was performed from 30 to 600 °C with a ramp of 10 °C/min under a nitrogen atmosphere (200 mL/min) in order to evaluate the thermal stability of CSW*.*

Differential scanning calorimetry (DSC) analysis was carried out in a DSC-Q200 instrument (TA Instruments, New Castle, Delaware, DE 19720, USA) under a nitrogen atmosphere. Samples of 6–10 mg were heated from −25 to 210 °C at a heating rate of 10 °C/ min (1st heating scan), hold 2 min at 210 °C to erase thermal history, cooled to −25 °C at a cooling rate of 10 °C/min (cooling scan) and heated again from −25 to 210 °C at a heating rate of 10 °C/min (2nd heating scan). Glass transition temperature (*T*_g_), melting temperature (*T*_m_) and cold crystallization temperature (*T*_cc_) values were determined.

### 2.6 Morphological Characterization

The morphological structure of 10 extracts was investigated by field emission scanning electron microscopy (FESEM, Supra 25-Zeiss, Oberkochen, Germany). Powder was deposited on conductive adhesive, gold sputtered, and visualized.

### 2.7 Mechanical Characterization

Tensile properties of PLA and PLA/CSW films have been measured according to the UNI ISO 527 standard method. Strips of each film were cut with a test dimension of 150 × 10 mm and conditioned for 48 h at (23 ± 2) °C and 50% RH before tests. The film strips were mounted on an LR30K universal electronic dynamometer by LLOYD Instruments (Segensworth West, Foreham, UK) with a 50 N load cell, and the initial gap between the clamps was set to 100 mm. Films were stretched at a cross-head speed of 5 mm/min. The tensile strength, elongation at break, and elastic modulus were determined. Measurements were performed on at least five replicates.

### 2.8 Transparency

The transparency of different PLA films was investigated by UV–vis spectroscopy in the range of 250–900 nm (Cary 4000, SpectraLab Scientific Inc., Markham, ON, Canada) at room temperature, employing a scan speed of 240 nm/min. Transmittance curves were obtained for each material.

### 2.9 Color Analysis

The color parameters of PLA films were determined by using a spectrophotometer (CM-2300d Konica Minolta, Japan). Data were acquired according to the SCI 10/D65 method, whereas CIELAB color variables, as defined by the Commission Internationale de l′Éclairage (CIE 1995), were utilized. The different formulations obtained were positioned on a white standard plate, and L*, a*, and b* parameters were analyzed. L* value ranges from 0 (black) to 100 (white); a* value ranges from −60 (green) to 60 (red); and b* value ranges from −60 (blue) to 60 (yellow). Samples were evaluated in triplicate, and three measurements were taken at random locations on each of the films studied. The total color difference ΔE* and gloss parameters were calculated as indicated in eq 1:

$$\Delta E^{*}=\sqrt{(\Delta L^{*}{)}^{2}+(\Delta a^{*}{)}^{2}+(\Delta b^{*}{)}^{2}}$$ 1

### 2.10 Antioxidant Activity of Films

The radical scavenging activity of PLA-based films was determined by using a spectroscopic method according to the literature.^24,25^ The different PLA films (0.1 g) were cut into small pieces and immersed in 2 mL of methanol for 24 h at RT. An aliquot of methanol extract (0.25 mL) was mixed with 1.75 mL of 2,2-diphenyl-1-picryl-hydrazyl radical (DPPH) in methanol (50 mg/L). The mixture was allowed to stand at RT for 60 min. The absorbance was measured at 517 nm using a UV spectrometer (Lambda 35). The DPPH mixture solution of methanol extracted from neat PVA was used as a control. DPPH radical scavenging activity (RSA) was measured according to eq 2:

$$\mathrm{RSA}\;(\%)=\frac{A_{\mathrm{Control}}-A_{\mathrm{Sample}}}{A_{\mathrm{Control}}}\times 100$$ 2

where *A*_sample_ is the absorbance of the sample, and *A*_control_ is the absorbance of the control.

### 2.11 Overall Migration Analysis

The overall migration analysis of PLA-based films was carried out in triplicate in simulant A (10% (v/v) ethanol/water solution) according to the current legislation Commission Regulation (EU) No. 10/2011. The analysis was performed to simulate the behavior of polymeric-based films in contact with a food simulant. Rectangular strips of 20 cm^2^ in 20 mL of food simulant were used.^26^ The samples were kept in a controlled chamber at 40 °C and removed after 10 days according to EN-1186 standard, and the simulant was evaporated in dishes and dried at 105 °C for 2 h in an oven. The residues were weighed with an analytical balance (Sartorius ATILON, Gottingen, Germany) with ± 0.01 mg precision, and the migration values in mg kg^–1^ of each simulant were determined.

### 2.12 Disintegration Test of Polymeric Packaging Material under Composting Conditions

The disintegration test under composting conditions of PLA-based films was evaluated following the UNI-EN ISO 20200:2016 Normative. The synthetic solid waste was prepared by the mixture of sawdust, rabbit food, mature compost, corn starch, sugar, corn oil, and urea, maintaining the water content around 50%. The synthetic solid waste was transferred to perforated boxes to ensure gas exchange between the inner atmosphere and the outside environment. Every day, the solid waste was softly mixed to ensure aerobic conditions during the test. On the other hand, squares of material (2.5 cm × 2.5 cm) were dried at 40 °C for 24 h and subsequently weighed. The samples were put into meshed plastic bags to ensure free contact with the solid waste and avoid the loss of small fragments during the test. The bags were buried in the synthetic solid waste at 4–6 cm depth and incubated at 58 °C and 50% relative humidity. The bags in contact with the synthetic solid waste were recovered after 1, 3, 7, 10, and 14 days. The samples were washed and dried at 40 °C for 24 h and then weighed. The degree of disintegration (Dis) of each formulation was calculated by eq 3.

$$\mathrm{Dis}\;(\%)=\frac{m_{i}-m_{r}}{m_{i}}\times 100$$ 3

where *m*_i_ and *m*_r_ are the initial and residual dry weights of the materials, respectively.

The physical changes of the samples during the test were analyzed through visual comparison by photographs.

### 2.13 Antibacterial Activity of PLA-CSW Films

The antimicrobial activity of each film was evaluated using both the disk diffusion method and the dilution method. For both assays, the inoculum of bacteria was prepared following the procedures described in paragraph 2.3. In the disk diffusion assay, plates were spread with 0.1 mL of the inoculum solution. Then, 10 mm diameter film disks were placed on the seeded plates. The plates were incubated at 37 ± 1 °C for 24 h, after which the diameter of the growth inhibition zone was measured.

To assess antimicrobial activity in liquid media, the method proposed by Llana-Ruiz-Cabello et al. was employed with slight modifications.^27^ Briefly, ten sterile strips (10 mm × 50 mm) of active or control films were placed in a test tube with 6 mL of saline solution and 1 mL of inoculum solution. The tubes were incubated for 24 h at 37 ± 1 °C for *S. aureus*, *E. faecalis*, *L. monocytogenes*, and 30 ± 1 °C for *B. cereus*. Additionally, a control test was conducted without film strips to determine the effective concentration of the bacterial inoculum. Plate count agar (PCA) was utilized for counting, using organism-specific media and growth conditions. PCA results were converted into logarithmic values. All determinations were performed in triplicate.

### 2.14 Statistical Analysis

Data provided from antimicrobial activities of PLA-CSW films were presented as mean ± standard deviation. To assess differences among factors and levels, a one-way analysis of variance (ANOVA) was conducted with the significance level set at *p* = 0.05. Mean comparisons were performed using the Dunnett test and Tukey’s HSD test. Pearson correlation was applied to estimate the relationship between bacterial growth and CSW concentration in active films. Data analysis was carried out using RStudio Desktop (version 2023.13.0 + 386, Posit Software, PBC, Boston, MA, USA), and graphics were created with the “ggplot2” package.

## 3 Results and Discussion

### 3.1 HPLC-DAD-MS Analysis of *Castanea sativa* Mill. Wood (CSW) Extract

*Castanea sativa* Mill. wood (CSW) extract was obtained from an aqueous solution originating from the production process of medium-density fiberboard (MDF) panels, which requires the removal of tannins from the wood before glue application, allowing the panels to be shaped before hardening. Therefore, the chestnut aqueous extract is a byproduct of wood processing. The extract was obtained through a sustainable and eco-friendly process based on hot water extraction, followed by fractionation and concentration using membrane technology, as previously described.^21^ It is applied in various sectors, including tanning, textiles, agronomy, livestock farming, and food and nutraceuticals. The chemical characterization of the CSW extract was carried out by HPLC-DAD-MS analysis. This technique, mostly employing reverse stationary phases, is useful for qualitative and quantitative analyses of polyphenols due to their polar nature. Comparison of chromatographic, spectrophotometric, and spectrometric data with those of analytical standards and literature data allows to characterize the single compounds belonging to the different phenolic subclasses, distinguishing the different isomers, which often remains challenging.^19,28–31^ Figure 1 shows the chromatographic profile recorded at 254 nm (a) and 280 nm (b); Table 1 reports, for each of the identified compounds, wavelengths of UV–vis maximum absorbance, ESI-MS molecular ions (*m*/*z*) in negative ionization mode, and the quantitative data.

**Figure 1:**
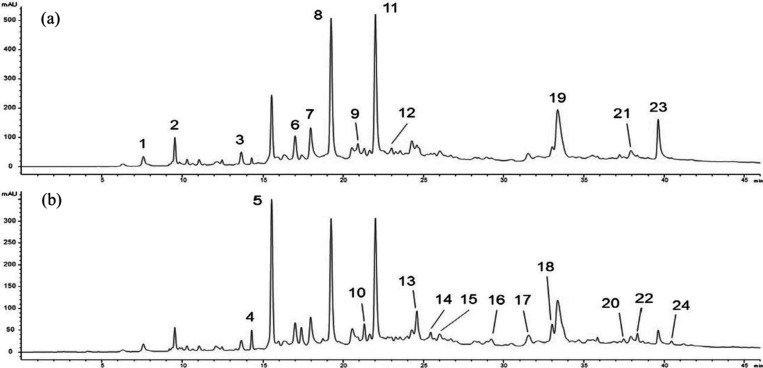
Chromatographic profile of *Castanea sativa* Mill. wood (CSW) extract recorded at 254 (a) and 280 nm (b). The identified peaks are numbered according to Table 1. 1. Vescalin; 2. Castalin; 3. Pedunculagin I; 4. Monogalloyl glucose; 5. Gallic acid; 6. Vescalagin/Castalagin pentoside I; 7. Vescalagin/Castalagin pentoside II; 8. Vescalagin; 9. Dehydrated tergallic-C-glucoside I; 10. Digalloyl glucose I; 11. Castalagin; 12. Dehydrated tergallic-C-glucoside II; 13. Digalloyl glucoseII; 14. Digalloyl glucose III; 15. Digalloyl glucose IV; 16. Trigalloyl glucose I; 17. Trigalloyl glucose II; 18. Trigalloyl glucose III; 19. *O*-Galloylated vescalagin/castalagin; 20. Tetragalloylglucose I; 21. Valoneic acid dilactone; 22. Tetragalloyl glucose II; 23. Ellagic acid; 24. Pentagalloylglucose.

**Table 1:** Quali-Quantitative HPLC-DAD-MS Analysis of *Castanea sativa* Mill. Wood (CSW) Extract^a^

peak	compound	λ_max_ (nm)	[M-H]^−^ (*m*/*z*)	mg/g	SD%
1	vescalin	246, 276 sh	631	2.8	4.3
2	castalin	246, 280 sh	631	3.2	4.2
3	pedunculagin I	258, 378 sh	783	5.7	4.0
4	monogalloyl glucose	274	331	4.9	2.0
5	gallic acid	272	169	27.3	1.9
6	vescalagin/castalagin pentoside I	240, 275 sh	1065	7.7	2.9
7	vescalagin/castalagin pentoside II	240, 275 sh	1065	12.2	2.9
8	vescalagin	245, 280 sh	933	35	3.0
9	dehydrated tergallic-C-glucoside I	250, 374	613	1.39	3.5
10	digalloyl glucose I	274	483	8.0	2.4
11	castalagin	245, 280 sh	933	38	3.1
12	dehydrated tergallic-C-glucoside II	250, 374	613	1.11	4.1
13	digalloyl glucose II	274	483	15.8	2.2
14	digalloyl glucose III	274	483	4.8	2.3
15	digalloyl glucose IV	274	483	4.5	2.6
16	trigalloyl glucose I	276	635	4.1	3.1
17	trigalloyl glucose II	276	635	12.6	3.0
18	trigalloyl glucose III	276	635	9.5	3.2
19	*O*-galloylated vescalagin/castalagin	220, 280 sh	1086	27.7	2.0
20	tetragalloyl glucose I	276	787	3.3	3.3
21	valoneic acid dilactone	258, 366	469	1.32	2.3
22	tetragalloyl glucose II	276	787	5.9	4.0
23	ellagic acid	254, 370	301	4.2	1.3
24	pentagalloyl glucose	274	939	3.7	3.0
	total polyphenols			245	2.7

a The results are expressed as mg per gram of dry extract; wavelengths (nm) of maximum UV–vis absorbance, molecular ions (m/z), and percentage standard deviations (SD%) are reported for each of the quantified compounds and for total polyphenols.

In the CSW extract, total polyphenols were 245 ± 6.6 mg/g. They consist mainly of gallic acid (27.3 ± 0.52 mg/g), together with hydrolyzable tannins as vescalagin and castalagin (35 ± 1.0 and 38 ± 1.2 mg/g, respectively, Figure 2). Gallic acid units are present also as mono- up to pentagalloyl glucose or bonded to each other through C–C bonds in hexahydroxydiphenoyl (HHDP) or nonahydroxytriphenoyl (NHTP) groups, like in vescalin/castalin and vescalagin/castalagin. Vescalin and castalin (molecular weight, MW = 632 g/mol) are derived from vescalagin and castalagin (MW = 934 g/mol) by hydrolysis, and they can yield dehydrated tergallic-C-glucoside (MW = 614 g/mol) after dehydration. Vescalagin and castalagin accounted, respectively, for 14.3 and 15.5% w/w on total polyphenols (35 ± 1.0 and 38 ± 1.0 mg/g), while vescalin and castalin are present in low amounts (1.1 and 1.3%). Compounds **6** and **7** are identified as vescalagin or castalagin pentosides (MW = 1066 g/mol) and accounted together for 8.1% of the total polyphenols. Vescalagin, castalagin, and their derivatives, except for dehydrated tergallic-C-glucoside (having a similar UV–vis spectrum to that of ellagic acid), are difficult to distinguish based on their UV–vis absorption profiles, which only indicate the presence of the HHDP- and NHTP- units. In this case, the respective ESI-MS data and chromatographic retention times were used to identify the individual compounds, comparing them to those previously reported in the literature.^21,22^ The presence of hydrolysis compounds and the lack of higher molecular weight tannins typical of CSW extract could be due to the high temperature during extraction and to the productive process.^21^ Compound **21**, tentatively identified as valoneic acid dilactone, has also been hypothesized to be a hydrolysis derivative of tannins present in chestnut extracts.^28,31^ Ellagic acid was present in a low amount (4.2 ± 0.2 mg/g).

**Figure 2:**
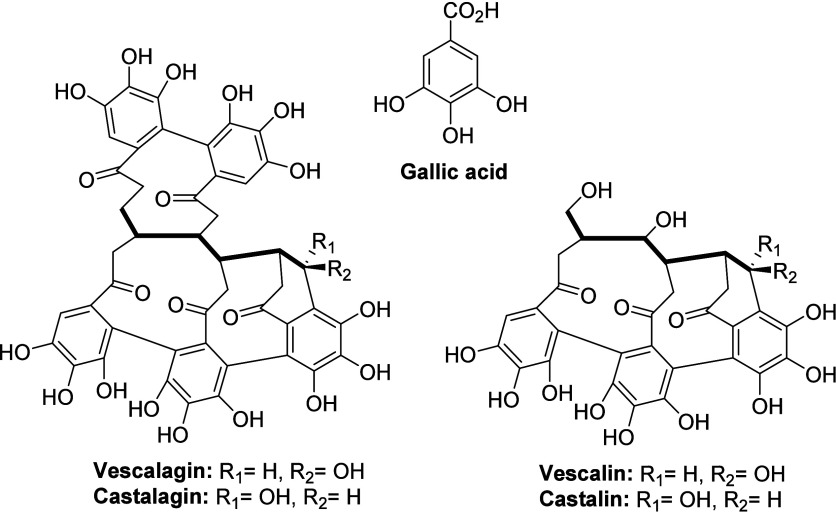
Chemical structures of the main phenolic compounds found in the CSW extract.

### 3.2 Morphological and Thermal Characterization of CSW Extract

The results shown in Figure 3a revealed a range of particle sizes, with the deviations observed that can be attributed to the irregular shape and nonspherical nature of the particles. The purification processes can alter the composition and molecular weight distribution of the tannins, affecting their ability to aggregate and form particles of varying sizes.^32^ The thermal decomposition of CSW starts with the evaporation of the adsorbed water from 50 to 170 °C, as the DTG curves indicate in Figure 3b; above that temperature, thermal decomposition of the organic components in the range 170–400 °C can be observed due to the presence of polysaccharides, which start degrading at a lower temperature than the polyphenolic compounds. A significant degradation occurs at 250 °C, due to the decarboxylation of the R-COOH groups with the release of carbon dioxide. A second peak at around 300 °C suggests that gallic acid derivatives may require higher temperatures to completely decarboxylate. The late degradation starts at 400 °C and weight loss is related to the oxidation of the residual carbon.^33,34^ Above 400 °C, charring reactions take place with the release of small molecules.^34,35^

**Figure 3:**
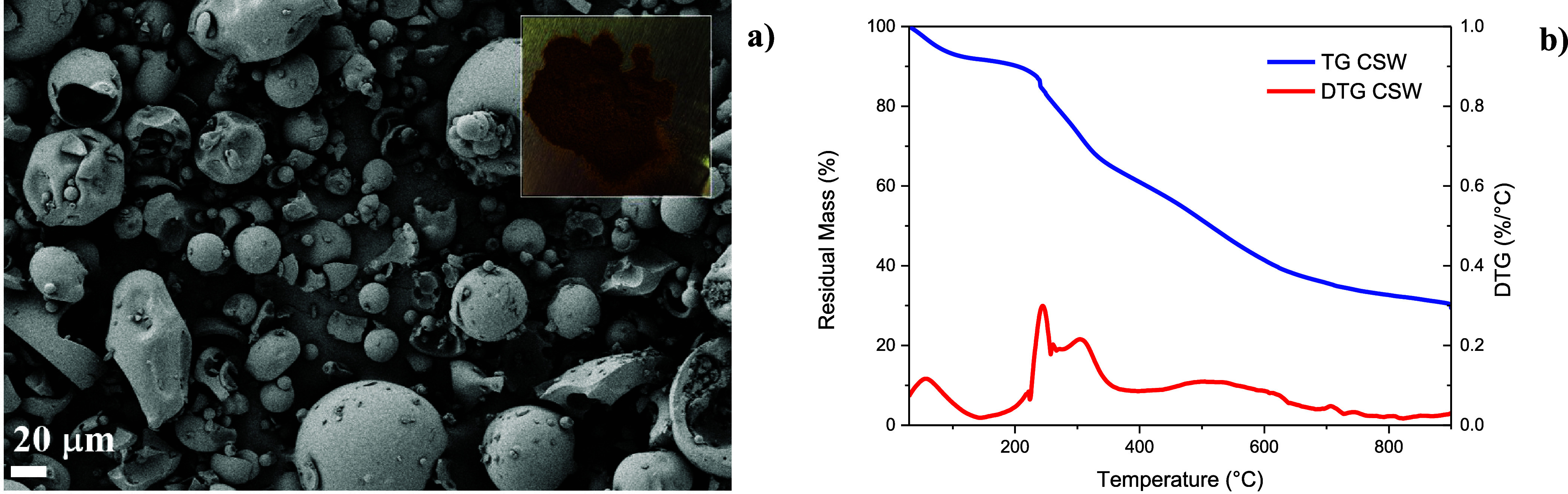
FESEM image (a) and TG/DTG curves (b) for the CSW extract.

### 3.3 Screening of Minimum Bactericidal Concentration (MBC) of CSW Extract against Gram-Positive and Gram-Negative Bacteria

Before formulating the active materials, the antibacterial properties of the CSW extract were screened against a range of Gram-positive and Gram-negative bacterial strains using a dilution method. The results reported in Table 2 revealed notable efficacy against Gram-positive bacteria, while no activity was observed against Gram-negative strains. Specifically, the MBC of the CSW extract was found to be 0.50% for *Staphylococcus aureus* ATCC 25923 and 1% for *Listeria monocytogenes* ATCC 13932, *Bacillus cereus* ATCC 11778, and *Enterococcus faecalis* ATCC 19433. The antibacterial effect against Gram-positive bacteria aligns with findings from previous studies on *Castanea sativa* extracts and byproducts.^36^ This activity is likely driven by key phenolic compounds, including gallic acid, vescalagin, and castalagin, which disrupt bacteria's cell wall integrity by interfering with peptidoglycan assembly, consequently promoting cell death.^37^ Conversely, no bactericidal activity was detected against Gram-negative bacteria, including *Escherichia coli* ATCC 25922, *Salmonella typhimurium* ATCC 14028, and *Pseudomonas aeruginosa* ATCC 27853. These findings, consistent with previous observations, could be mainly related to the presence of an outer membrane in Gram-negative bacteria that acts as a protective barrier against many antimicrobial compounds.^38^ Nevertheless, the results of antimicrobial tests can vary significantly depending on the chosen bacterial strain.^11,39^

**Table 2:** Qualitative Screening on Minimum Bactericidal Concentration (MBC) of CSW Extract at Different Concentrations (%v/v) against Gram-Positive and Gram-Negative Bacterial Strains

	CSW extract		
bacterial strain	1.0%^a^	0.5%^a^	0.1%^a^
*Staphylococcus aureus* ATCC 25923	-	-	+
*Listeria monocytogenes* ATCC 13932	-	+	+
*Bacillus cereus* ATCC 11778	-	-	+
*Enterococcus faecalis* ATCC 19433	-	+	+
*Escherichia coli* ATCC 25922	+	+	+
*Salmonella typhimurium* ATCC 14028	+	+	+
*Pseudomonas aeruginosa* ATCC 27853	+	+	+

a (-) Absence and (+) presence of bacterial growth

### 3.4 UV–Vis Characterization and Visual Observation of PLA-CSW Films

Figure 4 shows the UV–vis characterization and visual observation of PLA-CSW films. The photographic image displays the color and the aesthetic appearance of different polymeric films and the homogeneity of dispersion of CSW. The spectra from UV–vis analysis, performed for all polymeric films, confirmed the transparent nature of PLA film (transmittance of 95% at 600 nm); this high value was only slightly affected by the addition of CSW extract at different concentrations (transmittance at 600 nm: PLA_1CSW = 94.5%, PLA_3CSW = 93.5% and PLA_5CSW = 91.5%). A similar behavior was observed in literature employing also other active molecules.^40,41^ The transmittance trend was kept stable up to wavelength values of 600 nm, generating polymeric systems with high transparency in the wavelength range of 600–900 nm. Furthermore, the transparency for PLA loaded with 5 wt % of CSW decreased, due to the proper color of the natural ingredient.

**Figure 4:**
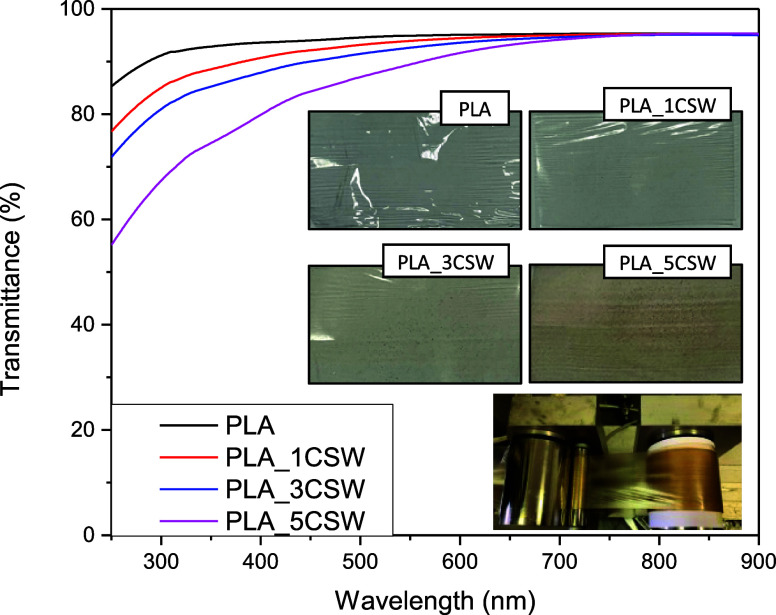
UV–vis analysis and visual image of PLA and PLA-CSW films.

The produced polymeric systems exhibited versatile behavior according to the wavelength range, since the addition of different concentration clearly affected the absorption of UV light (250–600 nm), especially by using the highest concentration of extract (5 wt % % with respect to polymeric content). Precisely, the addition of CSW at 5 wt % limited the transparency in the UVA region (315–400 nm) and increased the protection in the UVB (280–315 nm) region (lower transmittance in the UVB region compared to UVA wavelengths). This behavior acts positively, improving the shelf life of light-sensitive foods. Due to their physical, dimensional, and colorimetric properties, the extract hindered the passage of light radiation of specific wavelengths that could damage the packaged products and maintain at the same time the transparency.

### 3.5 Colorimetric Analysis

The colorimetric data of PLA-based films are summarized in Table 3. The colorimetric and gloss analysis allowed evaluation of the effect and the influence of CSW extract on the optical and aesthetic appearance of the polymeric films. Neat PLA film is characterized by a high lightness value (L* = 99.34 ± 0.05) compared to CSW-loaded films. The value highlights the transparency of the polymeric matrix, while a slight reduction of the lightness is registered for polymeric systems loaded with CSW as a coherent consequence of transparency reduction. This effect can be explained by the light-sensitive absorption of polyphenols.^42^ The b* values are positive for all formulations and indicate the chromatic tendency of these films to take on shades of brownish colors (see also the visual image of different produced films reported in Figure 4). The increase in concentration significantly contributes to the modification of the ΔE* values. The highest ΔE* value for PLA films has been registered for PLA_5CSW (ΔE* = 12.89 ± 0.38). The presence of the extract influences the aesthetic quality of PLA-based films; this aspect is evident when analyzing the gloss value that is reduced as the concentration of the extract increases.^10^

**Table 3:** Color Coordinates of PLA and PLA/CSW-Based Films

formulation	L*	a*	b*	gloss	ΔE*
PLA	99.34 ± 0.05	0.07 ± 0.01	0.05 ± 0.01	243 ± 2	--
PLA_1CSW	98.35 ± 0.20	0.12 ± 0.01	1.47 ± 0.12	232 ± 2	1.75 ± 0.21
PLA_3CSW	94.92 ± 0.33	0.97 ± 0.06	5.05 ± 0.13	175 ± 4	6.76 ± 0.32
PLA_5CSW	90.41 ± 0.49	2.55 ± 0.15	8.98 ± 0.14	122 ± 1	12.89 ± 0.38

### 3.6 DSC Analysis

DSC curves of PLA and PLA_CSW films obtained during the second heating process are presented in Figure 5. Three transformations comprised by DSC data were observed and analyzed: enthalpy relaxation corresponding to glass transition (about 60 °C), cold crystallization (near 109 °C), and melting of the polymer matrix (168 °C). It is noticeable that the incorporation of chestnut extract does not significantly influence the glass transition temperature determined by DSC. Pure PLA reveals low crystallization ability; therefore, macromolecular chains are unable to arrange completely during rapid cooling. No variations in cold crystallization temperatures were detected for PLA and its composite film, so the extract did not significantly affect this transition, as well as the melting behavior.^43^

**Figure 5:**
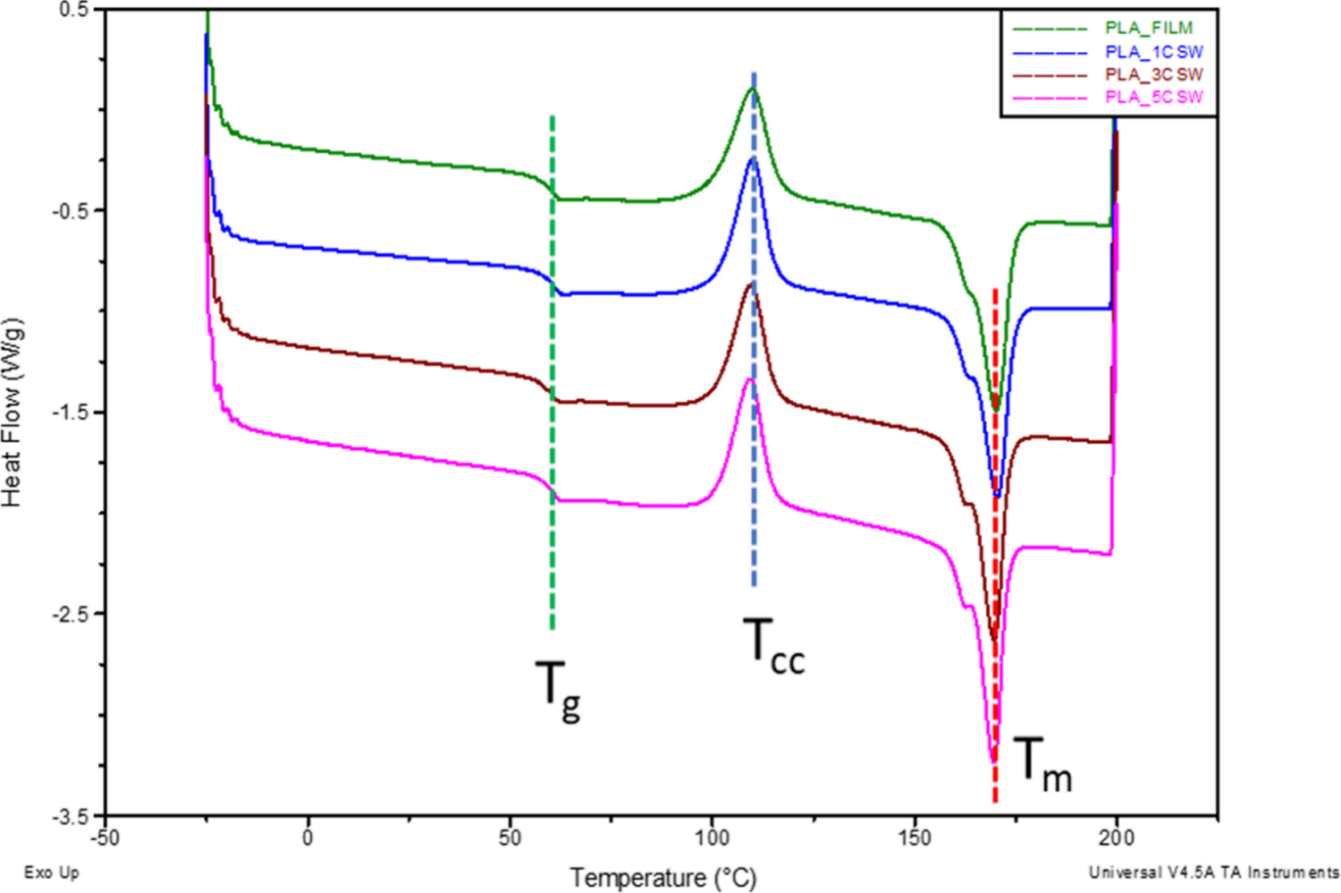
DSC curves (2nd heating scan) of PLA- and PLA/CSW-based films.

### 3.7 Antiradical Activity

Antiradical activity is one of the most important properties required for polymeric devices used in direct contact with foods. The ability to reduce the oxidative effect of food is a great advantage for foodstuff storage during shelf life. This is possible thanks to the use of active molecules, such as polyphenols. This active effect of polymeric systems is based on a migration process of active ingredients integrated in packaging materials into the foodstuff.^44^ The emerging problem of food waste in industrialized countries and the high demand for food products with long shelf life encouraged the development of active food packaging systems, and this issue has been addressed by employing active phenolic ingredients in packaging. The radical scavenging activity of polymeric systems is estimated by determining the scavenging activity of methanol migration of extracted substances against the DPPH radical. The DPPH radical scavenging capacity is an essential methodology to determine the antioxidant property.^45^ Figure 6 summarizes the antioxidant effects of migrated substances. Neat PLA is selected as the control. In detail, Figure 6a shows the monitoring of the absorbance band at 517 nm for migrating substances from PLA-based formulations immersed in methanol for 24 h, RSA (%) values, and color variation of the DPPH methanolic solutions. Figure 6b summarizes the effect against radical scavenging activities induced by the presence of the CSW extract in polymeric systems. In general, the polyphenols intercept and reduce the effect of the free radical oxidation chain by donating hydrogen from the phenolic hydroxyl groups.^25^ Chestnut skin extracts proved to have efficient antioxidant properties, specifically for lipid peroxidation.^46^ The incorporation of CSW guarantees clear antiradical scavenging activity according to the literature.^47^ The polymeric formulations loaded with 3 and 5 wt % CSW show similar values of antioxidant capacity (see Figure 6), likely because the system already saturates with 3% wt.

**Figure 6:**
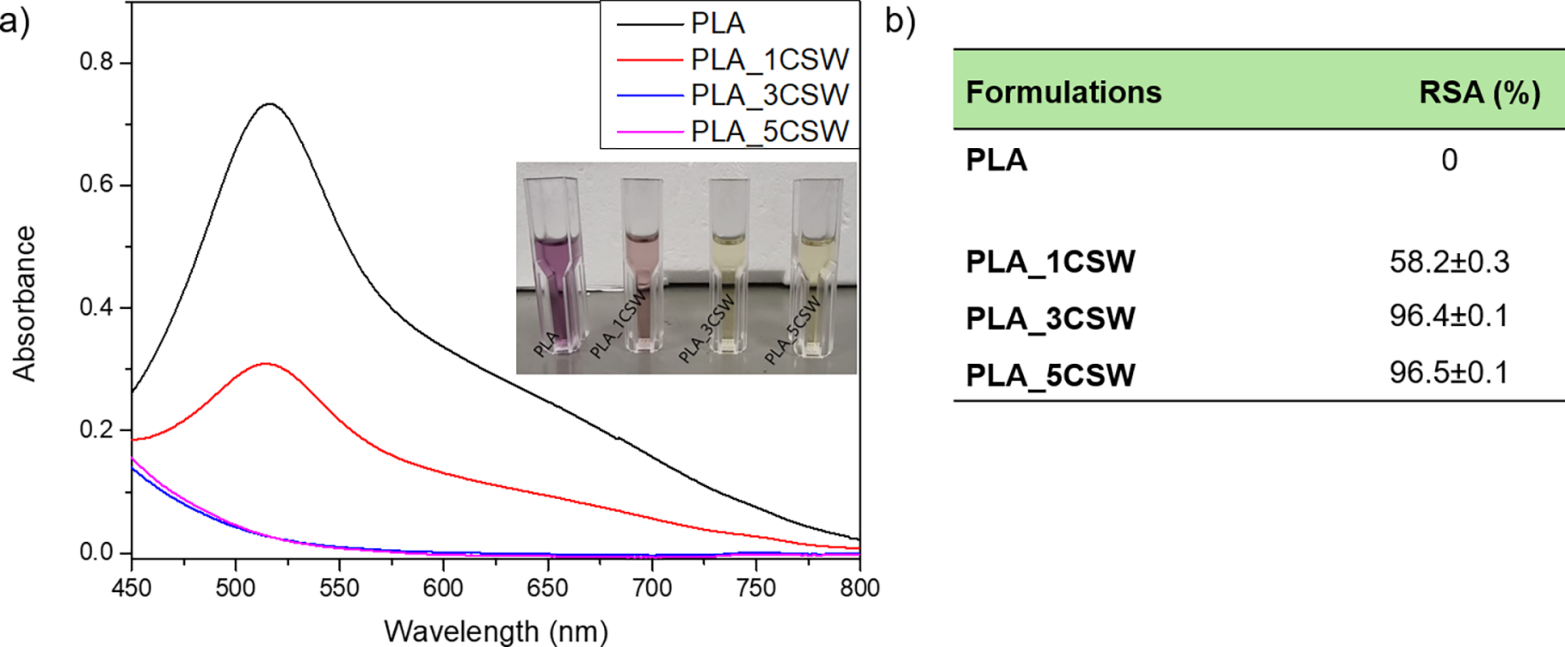
Antioxidant activities of migrating substances for PVA films immersed directly in the methanol solution for 24 h: monitoring of the absorbance band at 517 nm and color variation of the DPPH methanol solution (a) and RSA (%) values (b).

### 3.8 Overall Migration Test

Overall migration tests in aqueous simulant ethanol 10% (v/v) (simulant A) were performed to determine the total amount of nonvolatile compounds that could pass from the plastic material to aqueous food, which must be lower than the overall migration limit required by the current normative (10 mg/dm^2^) for food packaging materials.^26^ Figure 7 summarizes the migration levels for all of the tested systems. The obtained results demonstrate the potential applicability of polymeric systems in contact with fresh aqueous-based foods. It should be noted that the highest migration value, achieved by the PLA_5CSW-based formulation, is below the regulatory limit.

**Figure 7:**
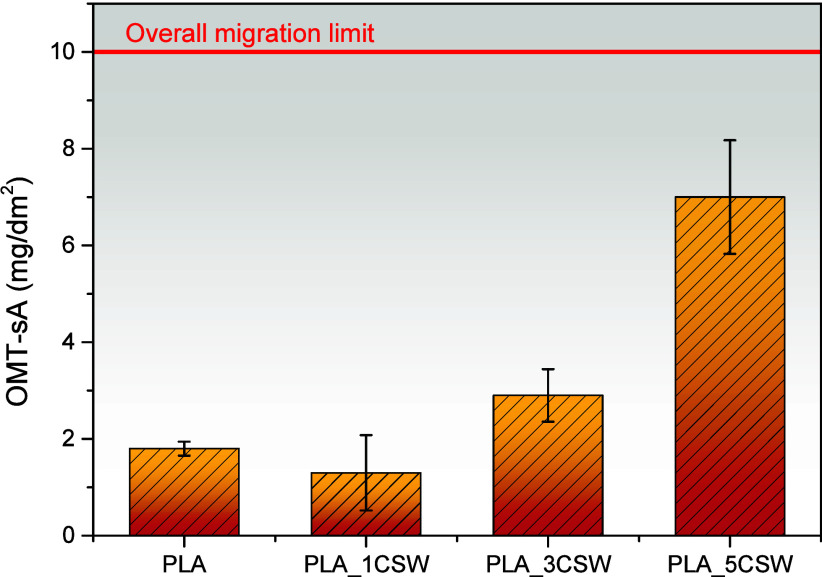
Overall migration data in ethanol 10% (v/v) for PLA- and PLA/CSW-based films. The dashed line indicates the overall migration limit (OML).

### 3.9 Disintegration in Compost

The disintegration in compost conditions is a decomposition process of organic matter carried out by microorganisms to carbon dioxide, heat, and water. Therefore, the result of this process is a soil enriched with nutrients, favorable for plant growth.^48^ The disintegration process of PLA starts with the sample fragmentation^49^ and the water diffusion through the polymeric matrix during the first two weeks. This phenomenon produces a nonenzymatic hydrolysis of the polymer with a reduction of molecular weight as an effect of random chain scissions through its ester groups.^50,51^ The fragmentation process leads to the formation of oligomers and lactic acid, assimilated by microorganisms to be converted to carbon dioxide and water.^52^ The disintegration in composting conditions can be affected by (i) the characteristics of the polymer (composition, molecular weight, crystallinity), (ii) the environmental conditions (humidity and temperature), and (iii) the presence of additives.^53^ Therefore, the effect of the addition of CSW during the disintegration in composting conditions was evaluated through macroscopic changes (size and color), disintegration level, and weight loss.

Figure 8 shows the visual changes (Figure 8a) and the disintegrability values (Figure 8b) that occurred during the composting of PLA films, taken out at different times of composting. The disintegrability value was evaluated in terms of weight loss as a function of time, in which the line at 90% of disintegration represents the limit point of disintegrability imposed by the ISO 20200; all the formulations reach a degree of disintegration exceeding 90% within 14 days in contact with compost soil. PLA material disintegrates very quickly with respect to the literature,^51,54^ in just 10 days. This phenomenon is also facilitated by the reduced thickness of the samples. The samples show evident visual fragmentation after 7 days of incubation. After only one day of incubation, the samples start to change their appearance: polymeric formulations start to appear white, and this effect is more evident after 3 days in composting conditions. The whitening color and opacity effect are attributed to a change in the refractive index due to water absorption, with the formation of low molecular weight polymeric formulations.^55^ The presence of CSW for the antimicrobial effect delays the degradative phenomenon compared to PLA films. This effect is more evident for PLA_1CSW-based films because the low content of active ingredient ensures an inhibition of bacterial growth without compromising the roughness of polymeric films, as highlighted by the gloss value (Table 3).

**Figure 8:**
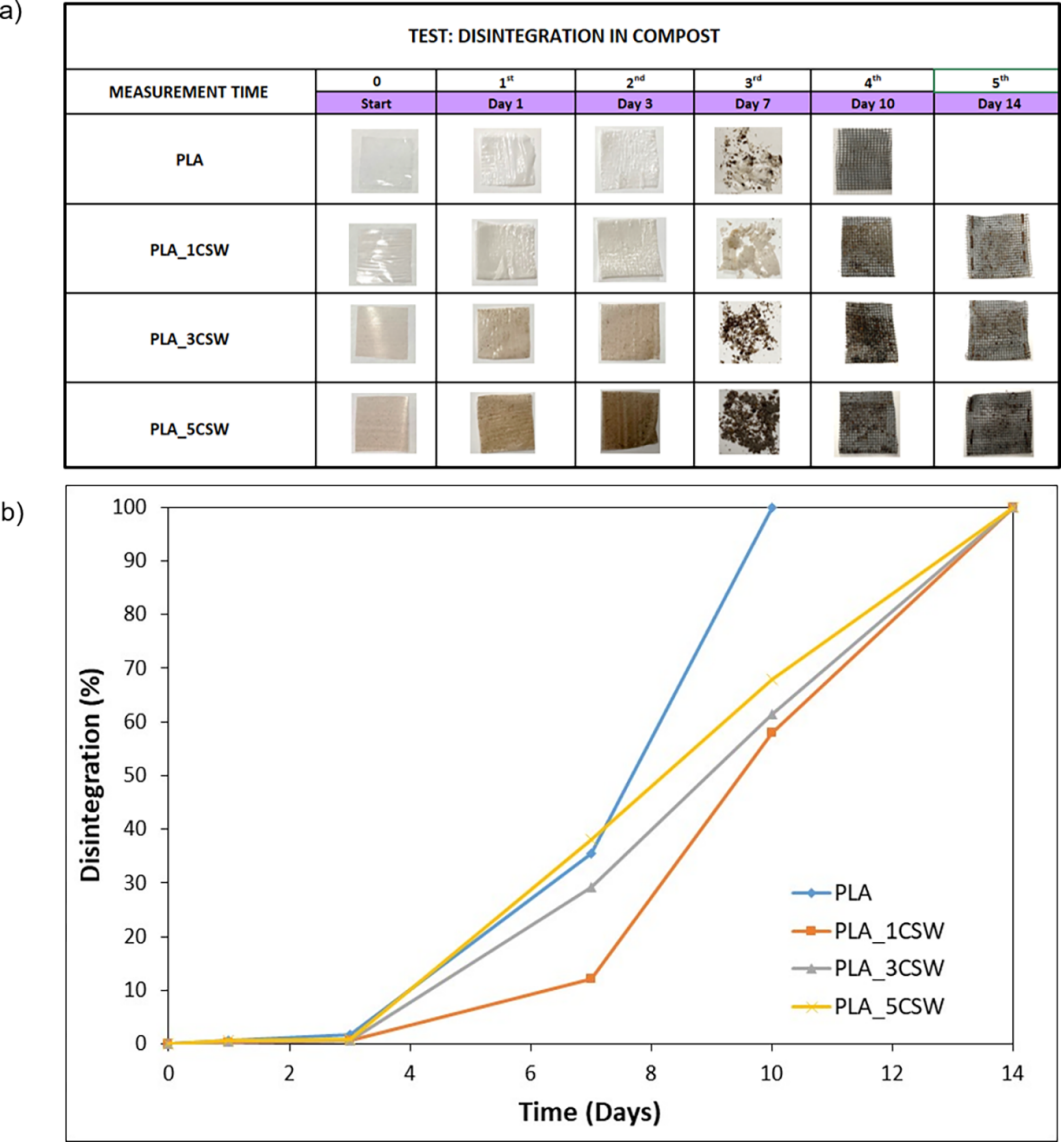
Visual appearance (a) and disintegration value % (b) for PLA- and PLA/CSW-based formulations at different stages of incubation in compost.

In Table 4, the results of mechanical characterization obtained by tensile tests show that the maximum tensile strength (σ_max_) of the reference PLA film assumes values in line with the reference literature;^56,57^ the composites containing 1, 3, and 5% wt of CSW show values of tensile strength (σ_max_) and Young’s modulus (*E*) that do not differ significantly from the reference matrix. The elastic modulus *E* of the composites is slightly improved compared to the pure PLA film, although without statistically significant variations. The main difference between the film of neat polylactic acid and the composites containing the chestnut extract occurs in the elongation at break (ε_b_); in particular, there is a reduction in the strain ε_b_, going from 20.1% of the pure matrix to values between 4.33 and 5.89%, without significant differences between the three composites.

**Table 4:** Results of Mechanical Characterization By Tensile Test According to ISO 527[Table-fn t4fn1]

sample	*E* (MPa)	σ_max_ (MPa)	ε_b_ (%)
PLA	1963 ± 94^a^	53.8 ± 1.7^a^	20.10 ± 3.57^a^
PLA_1CSW	2058 ± 57^a^	51.1 ± 1.5^a^	4.33 ± 1.32^b^
PLA_3CSW	2063 ± 54^a^	52.3 ± 1.0^a^	5.89 ± 1.57^b^
PLA_5CSW	2034 ± 41^a^	50.9 ± 1.6^a^	5.65 ± 1.70^b^

# Different superscripts (a−b) within the same column indicate significant differences among formulations values (*P* < 0.05).

The slight increase in the elastic modulus and the reduction in the elongation at break are related to the presence of CSW particles, which produce stress concentration centers that are able to generate fracture trigger points. This result is in line with the morphological analysis that highlighted the poor adhesion at the interface between CSW extract particles and the polymer matrix. The values of ultimate tensile strength are not influenced by the presence of CSW particles, since the maximum stresses are reached at the yield point that precedes the plastic elongation phase, as can be observed from the σ–ε curves (Figure 9a) representing the films. It can be deduced that the addition of bioactive particles rich in phenols does not modify the mechanical properties, except for a moderate reduction in the deformability of the composites, but without making them brittle (Figure 9b). Despite the decrease in strain, keeping the other mechanical parameters unchanged, the use of composites as bioactive packaging is not compromised; in fact, bioactive composites maintain adequate mechanical properties and show other advantages, such as antioxidant and fungal growth inhibitory effects.

**Figure 9:**
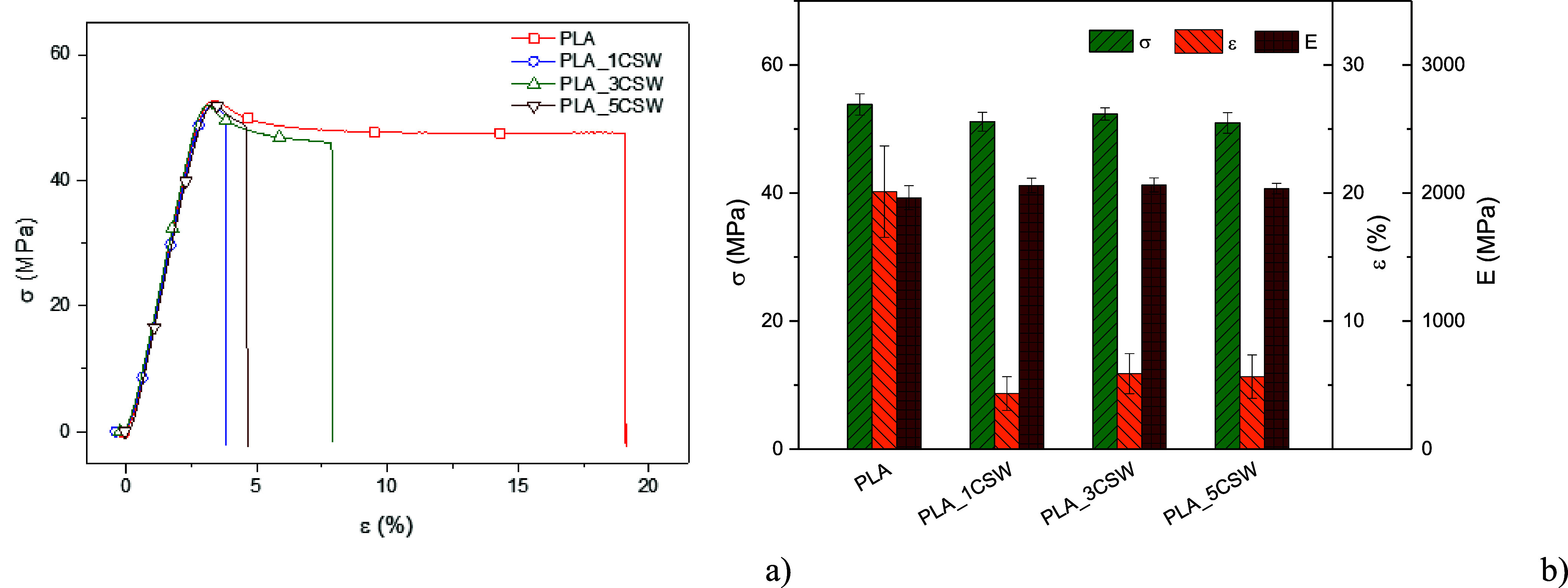
Stress–strain curves (a) and mechanical characteristic parameters obtained by the tensile test (b).

### 3.10 Antibacterial Activity of PLA-CSW Formulations

To the best of our knowledge, this is the first study reporting the antibacterial activity of an active sweet chestnut-PLA-based material. While no inhibitory zones were observed using the diffusion method, an absence of bacterial growth beneath the disk was noticed. This could suggest potential activity via direct contact. The lack of diffusion-based inhibition could be due to poor diffusion of the active compound from the PLA matrix under these testing conditions^27^ (Figure 10). On the other hand, the dilution method provided encouraging results (Table 5). A dose-dependent antimicrobial activity was observed against Gram-positive *Staphylococcus aureus* ATCC 25923, with a Pearson correlation of −0.96 between CSW concentration and bacterial growth. Specifically, PLA_3CSW film reduced bacterial CFU/mL by more than 2log while a total inhibition was observed with PLA_5CSW. These results suggest potential biomedical applications, where secondary metabolites such as tannic acid, epicatechin gallate, and gallic acid demonstrated the ability to prevent *S. aureus* adhesion on treated 3D-printed materials.^58^

**Figure 10:**
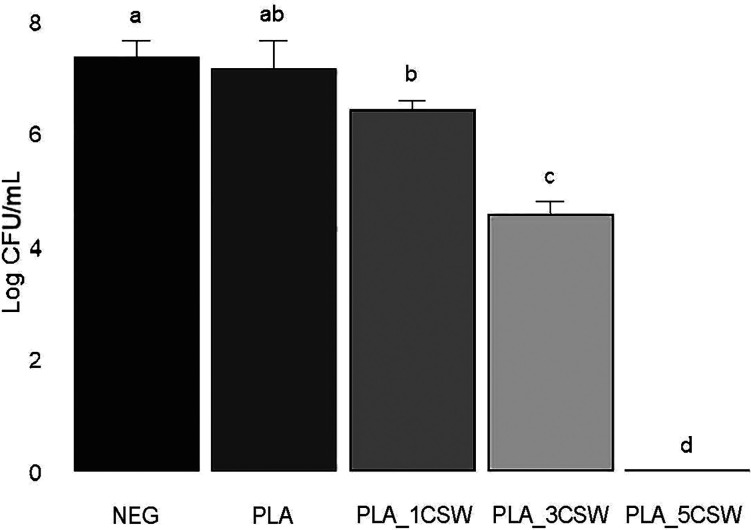
Antimicrobial activity of PLA_CSW material against *Staphylococcus aureus* ATCC 25923. Data expressed by mean ± SD (*n* = 3). Values with different letters are significantly different according to Tukey’s HSD test (*p* < 0.05).

**Table 5:** Antibacterial Activity of the PLA_CSW Films against Gram-Positive Strains^a^

	*Staphylococcus aureus* ATCC 25923	*Bacillus cereus* ATCC 11778	*Listeria monocytogenes* ATCC 13932	*Enterococcus faecalis* ATCC 19433
**film**	**CFU/mL (log)**	**CFU/mL (log)**	**CFU/mL (log)**	**CFU/mL (log)**
NEG	7.36 ± 0.28	6.78 ± 0.42	6.90 ± 0.40	7.10 ± 0.28
PLA	7.15 ± 0.51	6.92 ± 0.34	6.76 ± 0.40	7.05 ± 0.38
PLA_1CSW	6.42 ± 0.17*	6.79 ± 0.28	6.43 ± 0.49	6.84 ± 0.34
PLA_3CSW	4.54 ± 0.23***	6.79 ± 0.28	5.95 ± 0.66	6.27 ± 0.38
PLA_5CSW	0.00 ± 0.00***	1.23 ± 1.09***	6.24 ± 0.28	6.61 ± 0.41
ANOVA	*p* < 0.001	*p* < 0.001	*p* > 0.05	*p* > 0.05

a Data expressed by mean ± SD (*n* = 3). Dunnett’s test results comparing each test group to the negative control (NEG). Significant differences are indicated as follows: *p < 0.05, **p < 0.01, ***p < 0.001.

A similar effect was noticed with *Bacillus cereus* ATCC 11778, where the PLA_5CSW film almost completely stopped bacterial growth, demonstrating its strong antimicrobial effect at this concentration. However, lower concentrations of PLA_3CSW did not show significant inhibition. For *Listeria monocytogenes* ATCC 13932 and *Enterococcus faecalis* ATCC19433, the active films had no observable effect, regardless of the concentration. These unexpected outcomes, particularly compared to minimum bactericidal concentration (MBC) results for the CSW extracts, may be attributed to operational factors or film characteristics, such as the amount of CSW released.

Based on the literature data, the antibacterial activity of the PLA-CSW materials can be attributed to the release of CSW’s phenolics (e.g., gallic acid, vescalagin, and castalagin) in the aqueous environment during tests based on dilution methods. These compounds are known to disrupt the cell wall of Gram-positive bacteria by interfering during peptidoglycan assembly.^37^ The lack of activity against Gram-negative bacteria can be due to their outer membrane, which limits the access of antimicrobials.^38^ In addition, based on the results of the diffusion tests, no inhibition zones were observed due to the limited release of active compounds from a hydrophobic matrix such as PLA. However, the absence of bacterial growth directly beneath the film could suggest a potential contact-dependent effect.^25^ Future work could be focused on the optimization of the polymer matrix formula to enhance the controlled release of bioactive compounds.

## 4 Conclusions

This study successfully demonstrated the valorization of *Castanea sativa* Mill. wood byproducts within a PLA matrix, leading to the development of compostable and bioactive films consistent with the principles of the circular economy. The phenolic-rich extract obtained from chestnut wood residues, characterized by high levels of gallic acid, ellagic acid, and hydrolyzable tannins such as vescalagin and castalagin, exhibited remarkable antioxidant properties and significant antibacterial activity against Gram-positive bacterial strains. The incorporation of CSW extract into PLA via extrusion produced homogeneous and thermally stable materials up to 250 °C, with mechanical properties comparable to those of neat PLA. A moderate reduction in elongation at break was observed, related to the interaction between phenolic particles and the polymer matrix. The antioxidant activity of the films was found to be dose-dependent, reaching nearly 100% radical scavenging efficiency for PLA_3CSW, while PLA_5CSW showed strong antibacterial activity against *Staphylococcus aureus* and *Bacillus cereus*. In terms of application, overall migration tests confirmed the suitability of these materials for food-contact use with migration levels well below the European regulatory limit (10 mg/dm^2^). Moreover, all samples achieved over 90% disintegration within 14 days under controlled composting conditions, in accordance with ISO 20200:2016, confirming their full compostability. In a broader sustainability context, these findings highlight the feasibility of transforming agro-industrial waste into high-value, multifunctional materials. The use of CSW extract not only enhances the performance of PLA but also contributes to waste reduction and resource efficiency. Future research should focus on optimizing the dispersion of CSW extract within the polymeric matrix and elucidating the release dynamics of the active components to further expand potential applications in sustainable packaging and biomedical fields.
